# Prodromal stage and clinical features of late-onset schizophrenia and schizophrenia-like psychosis

**DOI:** 10.1192/j.eurpsy.2023.2130

**Published:** 2023-07-19

**Authors:** V. Pochueva, V. Sheshenin

**Affiliations:** 1 FSBSI Mental Health Research Center, Moscow; 2 FSBSI Mental Health Research Center, Moscow, Russian Federation

## Abstract

**Introduction:**

The early diagnostic of schizophrenia and other psychosis is very important for the early therapeutic interventions.

**Objectives:**

The aim is to describe the connection between the prodromal stage of psychosis and clinical features.

**Methods:**

74 patients with late-onset psychosis (mean age 64,33±9, 2 male; age of onset 55,3±11,2): late-onset schizophrenia (LOS) (n=49, mean age 63,0±8,47, age of onset 53,9±9,56), late-onset schizoaffective disorder (LOSaD) (n=17, mean age 62,4±6,5, age of onset 54,6±10,6, 2 male), late onset delusion disorder (LODD) (n=8, mean age 76,6±4,3, age of onset 65,2±17,0). Psychopathological, statistical methods were applied.

**Results:**

Allocated 4 types of prodromal stage – 1^st^ without psychopathological signs (n=24, 33%), 2^nd^ – with affective signs like disturbances of mood, anxiety (n=18, 24%), 3^rd^ – with paranoid signs like acute stress-related paranoid reactions without medication; 4^th^ - with schizoid signs with overvaluated ideas. In the 1^st^ group next syndromes prevailed: with secondary persecutory mood-congruent delusions (n=10, 41,7%); with auditory second-person pseudohallucinations with sistematyzed persecutory delusions (n=9, 37,5%); with only systematized persecutory delusions (n=1, 4,1%); with bizzarre delusions (n=3, 12,5%) and with polymorphic symptoms, include different hallucinations, catatonia disorders and with some oneiroid state signs (n=1, 4,1%). In this group 9 patients were diagnosed with LOS (37,5%); 12 patients with LOSaD (50%) and 3 patients with LODD (12,5%). The 2^nd^ group was presented with auditory second-person pseudohallucinations with sistematyzed persecutory delusions (n=5, 27.7%), with secondary persecutory delusions with delusion mood (n=11, 61%), with systemized persecutory delusional - 5.5% (n=1) and with catatonia (n=1, 5.5%). In this group 12 patients were diagnosed with LOS (66%), 5 patients with LOSaD (28%) and 1 patient with LODD (5.5%). In the 3^rd^ group these syndromes prevailed: with auditory second-person pseudohallucinations with sistematyzed persecutory delusions (n=7, 63%), with secondary persecutory delusions with delusion mood - in 2 cases (18.2%), with bizarre delusions - in 2 cases (18.2%). 12 patients were diagnosed with LOS (n=10.91%) and 1 patient with LODD (1.9%). The 4^th^ group was presented with auditory second-person pseudohallucinations with sistematyzed persecutory delusions (n=5, 23.8%), with secondary persecutory delusions with delusion mood (n=3, 14.3%), with bizarre delusions (n=6, 28.6%), with systemized persecutory delusions (n=1, 4.7%), with catatonia (n=2, 9.5%) and with polymorphic symptoms (n=4, 20%). 18 patients were diagnosed with LOS (85.7%) and 3 patients - with LODD (14.3%).

**Conclusions:**

There are different types of prodromal stage in late-onset psychosis that concluded with clinical features.

**Disclosure of Interest:**

None Declared

